# Low case notification rates of childhood tuberculosis in southern Ethiopia

**DOI:** 10.1186/s12887-015-0461-1

**Published:** 2015-10-01

**Authors:** Mesay Hailu Dangisso, Daniel Gemechu Datiko, Bernt Lindtjørn

**Affiliations:** Centre for International Health, Faculty of Medicine and Dentistry, University of Bergen Armauer Hansen Building, N-5012 Bergen, Norway; Sidama Zone Health Department, Hawassa, Ethiopia; Hawassa University, Hawassa, Ethiopia; Liverpool School of Tropical Medicine, Liverpool, UK

**Keywords:** Childhood tuberculosis, Childhood TB case notification, Treatment outcome, Sidama, Ethiopia

## Abstract

**Background:**

Childhood tuberculosis (TB) is a public health concern causing considerable mortality. However, control of childhood TB receives little attention. The control efforts could be inadequate because of challenges associated with difficulties in diagnosing the disease in children. Understanding the burden of the disease among children is important to assess the ongoing transmission of the disease in a community and improving TB control efforts. This study was carried out to assess TB case notification rates (CNRs) and treatment outcomes in children aged less than 15 years over a ten-year period.

**Methods:**

Data were collected from unit TB registers from all health facilities providing TB treatment in the Sidama Zone in Ethiopia. We analysed the CNRs and treatment outcomes by age category, gender, and place of residence. We used logistic regression analysis to identify factors associated with treatment outcomes and to control for confounding.

**Results:**

A total of 4,656 cases of children less than 15 years of age were notified as diagnosed and treated for TB, constituting 13 % of all notified TB cases in the study area. The mean CNRs per 100,000 children less than 15 years were 30 for all new cases of TB, 28 for rural cases, 67 for urban cases, 28 in boys, and 32 in girls. The proportions of treatment success were 82 % for new and 77 % for retreatment cases for the entire study period and increased to 93 % for new cases in 2012 (X^2^_trend_, *P* < 0.001). Children less than five years old had a lower treatment success [adjusted odds ratio (AOR) 0.64 (95 % CI, 0.52-0.80)] and higher deaths [AOR 2 (95 % CI, 1.27–3.12)]. The proportion of children who died during treatment among children in the less than 2-year-old age group was three times higher than children in the 2 year and above age groups [AOR 3.34 (95 % CI, 1.92–5.82)].

**Conclusion:**

The CNRs of childhood TB were low in Sidama. Children less than 5 years old had a higher proportion of deaths. Efforts need to be made to improve the diagnosis and treatment of TB among children.

**Electronic supplementary material:**

The online version of this article (doi:10.1186/s12887-015-0461-1) contains supplementary material, which is available to authorized users.

## Background

Childhood tuberculosis (TB) is a public health concern causing considerable mortality [1–3]. In 2013, approximately 550,000 new cases of TB and 80,000 deaths occurred in children who were HIV-negative [2]. However, childhood TB has been addressed inadequately in resource constrained settings due to challenges associated with difficulties in diagnosis and treatment of the disease [3, 4]. Lack of trained human resources to deliver the services, high cost related to training and to provision of services, and limited diagnostic facilities are some of the challenges for childhood TB diagnosis and control [5]. On the other hand, low case notification rates (CNRs) of TB in children could be because of underdiagnoses, underreporting, and due to the lower priority that the health systems give for children with TB compared to adults [6, 7]. In areas with poor access to diagnostic facilities and skilled personnel, children could suffer from the disease even without being diagnosed, or they are diagnosed late. Late diagnosis can contribute to considerable childhood deaths and to the transmission of the disease.

In Ethiopia, the National TB Control Program decentralized and expanded the directly observed treatment short course (DOTS) strategy. However, the existing TB control programs may have limitations in addressing childhood TB. The national TB prevalence survey of Ethiopia did not report the estimates of TB burden in children [8]. There is also limited information on age disaggregated case notification and treatment outcomes in routine surveillance system.

The burden of TB in children can serve as an indicator to assess effectiveness of TB control programs, and one of the indications for the ongoing transmission of the disease in a community [9]. Therefore, generating information from surveillance data is useful for understanding the burden of the disease in a community and such information could provide essential evidence for improving tuberculosis control [6, 7]. The objective of this study is to assess case notification rates and treatment outcomes of childhood TB over a ten-year period in the Sidama Zone in southern Ethiopia.

## Methods

### Study setting

The study was conducted in the Sidama Zone in southern Ethiopia, which is one of the most densely populated areas in Ethiopia with a population of over 3.4 million. Ninety-two per cent of the population live in the rural areas, and agriculture is the major livelihood of the community. Administratively, the Sidama Zone is divided into 19 woredas (districts) and two towns. There are 524 rural and 39 urban kebeles (the lowest administrative unit for about 5,000 people or 1000 households on average). In 2012, 114 health facilities provided TB treatment and 81 health facilities carried out sputum-smear examination. Ninety-five percent of the districts in the study area had at least one antiretroviral treatment (ART) centre. The demographic and health survey of Ethiopia estimated the adult HIV prevalence in the southern region of Ethiopia, where the present study was conducted, to be approximately 1 % [10]. In 2011, a community based intervention was implemented aimed at improving TB case finding and treatment outcomes [11]. In 2012, there were only two health facilities with radiographic services.

### TB diagnosis and treatment definition

We used the National TB control guideline of Ethiopia for diagnosis and treatment, case definition, and treatment outcomes [12].

Smear-positive pulmonary TB (PTB) is diagnosed with at least two positive initial sputum smears for Acid Fast Bacilli (AFB) by direct microscopy, or one positive smear for AFB by direct microscopy and culture positive for *Mycobacterium tuberculosis* or one positive smear for AFB by direct microscopy and radiographic abnormalities consistent with active TB as determined by a clinician. The laboratory keeps all positive and negative slides for external quality assurance. Quality assurance is performed regularly at the regional laboratory, and feedback is given to a reporting health facility. Previous studies reported a high specificity and good agreement of sputum-smear microscopy for AFB between peripheral and reference laboratories [11, 13].

Smear-negative pulmonary TB (PTB) is diagnosed when the patient presents with symptoms suggestive of TB, has at least three initial smear examinations negative for AFB, no response to antibiotics, repeat smear-negative and radiological abnormalities consistent with pulmonary TB, as well as a clinician’s decision.

Extra pulmonary TB (EPTB) is diagnosed by one culture-positive specimen from an extra pulmonary site or histopathological evidence from a biopsy, which is based on strong clinical evidence consistent with active EPTB by a clinician’s decision. However, most health facilities diagnose the disease based on a clinician’s decision because there are inadequate laboratory facilities for sputum culture or histopathology. The body sites for EPTB are lymph nodes, intestine, bone, kidney, central nervous system, and other organs.

A short-course treatment regimen is given in two phases with fixed-dose combination first-line anti-TB drugs [12]. The intensive phase treatment lasts for two months with ethambutol (E), isoniazid (H), rifampicin (R) and pyrazinamide (Z) followed by a continuation phase of six-month with ethambutol and isoniazid. Since 2011, the continuation phase has lasted for four months, and uses isoniazid and rifampicin.

“Cured” is smear-positive TB patient who is sputum smear-negative at, or one ‘month’ prior to the completion of treatment and on at least one previous occasion.

“Treatment completed” is a TB patient who completed treatment but for whom smear results are not available one month prior to the completion of treatment.

“Treatment success” is patients who was declared “cured” or “treatment completed”

### Data collection

The data were collected from August 2012 to February 2013 from all DOTS providing health facilities during 2003 to 2012. We collected unit TB registers from all health facilities in the study area and included the following variables: address of the patient, name of the health facility, age, sex, smear result, TB category, TB classification, intensive phase treatment drugs, year of treatment, date of treatment started, last date of treatment, and treatment outcomes. The treatment results included treatment completed, cured, defaulted (lost-to-follow up), died, transferred out, treatment failure, and unknown. We could not collect the data of nutritional status and HIV status of the children because the data were not recorded in the unit TB registers. Address of patients consisted of actual district and kebele of the patient at the time of diagnosis. Data entry personnel were university graduates with experience of data management. Training was given to data collectors and the data were double entered. We cross checked the data for the number of cases by year, facilities and districts as well as the correctness and consistency of information. The data were entered using Microsoft Access and exported to IBM SPSS (Statistics for Windows, Version 20.0. Armonk, NY: IBM Corp).

We supervised the data entry for completeness and consistency on regular basis. To ensure data quality we checked consistency and correctness of entered information with information in the TB registers. We did an exploratory analysis, looked for errors and corrected them from the registers. Number of cases and patient information entered by each year and health facility were checked with information in unit TB register page by page and year of treatment to ensure data completeness and accuracy.

### Statistical analysis

Case notification and treatment outcomes were computed for smear positive PTB, smear negative PTB and EPTB cases. We obtained each year’s population data for the study area from Central Statistical Agency of Ethiopia (CSA) [14] and computed case notification rates using under 15 years population for different years by age category as a denominator and notified cases as a numerator. We used IBM SPSS 20 for data analysis. Multivariate logistic regression analysis was performed to determine independent risk factors associated with treatment outcomes and to control for confounding.

### Ethical consideration

We obtained ethical clearance from the Regional Health Bureau of southern Ethiopia, in addition to a letter of support from the Sidama Zone Department of Health, to obtain information from all districts and health facilities. Personal identifiers of the cases were coded prior to analysis and medical records were kept in a secure place to help maintain the confidentiality of clinical information of cases.

## Results

A total of 4,656 cases of children less than 15 years of age were notified as diagnosed and treated for tuberculosis from 2003 through 2012, constituting about 13 % of cases of all age groups notified in the study area. Fifty-two percent (2,434 cases) of the cases were girls and 48 % (2,220 cases) were boys. Fifteen percent (719 cases) of the cases were less than five years old and 2,433 cases (53 %) were in the 10–14-year-age group. Of the cases, 4,087 (88 %) were from rural areas, 1,956 cases (42 %) were smear-positive PTB, and 1,308 (28 %) were smear-negative PTB cases (Table 1). The proportion of smear-positive PTB in the younger age group (less than 5 years) was lower than the proportions among 5–9 and 10–14 year-age groups (Table 2).

**Table 1:** General characteristics of the tuberculosis cases among children 0–14 years and all age groups in the Sidama Zone, 2003–2012

Characteristics	Childhood TB(Age 0–14 years)	Total notified cases (All age groups)	Proportion of childhood TB
	*N* (%)	*N* (%)	%
Sex			
Male	2,220 (48)	20,193 (54.5)	11
Female	2,434 (52)	16,867 (45.5)	14.4
Age category			
<5	740 (16)	-	-
5–9	1,430 (31)	-	-
10–14	2,486 (54)	-	-
Residence			
Urban	559 (12)	5,158 (14)	10.8
Rural	4,087 (88)	31,912 (86)	12.8
TB classification			
Pulmonary smear positive	1,956 (42)	22,545 (61)	8.7
Pulmonary negative	1,307 (28)	7,980 (22)	16.4
Extra pulmonary	1,379 (30)	6,464 (17)	21.3
Not mentioned	14 (0.3)	72 (0.2)	19.4
Patient category			
New	4,545 (98)	35,314 (95.3)	12.9
Retreatment	90 (1.9)	1,641 (4.4)	3.4
Transferred in	16 (0.3)	102 (0.3)	29.4
Not mentioned	5 (0.1)	13 (0)	38.4
Treatment outcomes			
Completed	2,586 (55.5)	16,161 (44)	16
Cured	1,187 (25.5)	14,205(38)	8.4
Lost-to-follow up	482 (10.4)	3,284 (9)	14.7
Died	140 (3.0)	1,202 (3.2)	11.6
Transferred	109 (2.3)	874 (2.4)	12.5
Treatment failure	9 (0.2)	92 (0.2)	9.8
Unknown	143 (3.1)	1,252 (3.4)	11.4
Year of treatment			
2003	367 (7.9)	2,341 (6.3)	15.7
2004	387 (8.3)	2,705 (7.3)	14.3
2005	369 (7.9)	2,774 (7.5)	13.3
2006	349 (7.5)	2,523 (6.8)	13.8
2007	483 (10.4)	3,296 (8.9)	14.7
2008	527 (11.3)	4,125 (11.1)	12.8
2009	433 (9.3)	3,499 (9.4)	12.4
2010	385 (8.3)	3,303 (8.9)	11.7
2011	662 (14.2)	5,643 (15.2)	11.7
2012	694 (14.9)	6,846 (18.5)	10.1
Total cases	4,656	37,070	12.6

The year of treatment was not mentioned for 15 cases for all age groups

**Table 2:** Characteristics childhood TB cases by TB classification in the Sidama Zone, 2003–2012

Characteristics	Smear positive *N* (%)	Smear negative *N* (%)	Extra pulmonary *N* (%)	Total^a^ *N* (%)
Number of TB cases (N)	1,956	1, 307	1,379	4642
Sex				
Girls	1092 (56)	653 (50)	681 (49)	2,426 (52)
Boys	863 (44)	654 (50)	698 (51)	2,215 (48)
Age category				
<5	130 (7)	362 (28)	244 (18)	736 (16)
5–9	494 (25)	453 (35)	478 (35)	1,425 (31)
10–14	1332 (68)	492 (38)	657 (47)	2,481 (53)
Residence				
Urban	178 (9)	193 (15)	198 (14)	569 (12)
Rural	1778 (91)	1114 (85)	1181 (86)	4,073 (88)
Patient category				
New	1902 (98)	1278 (98)	1359 (99)	4,539 (98)
Repeat	47 (2)	266 (1.2)	16 (1)	89 (2)
Transferred	7 (1)	1 (1)	4 (0.3)	12 (0.3)
Treatment outcomes				
Completed	419 (21)	1038 (79)	1128 (82)	2,585 (56)
Cured	1184 (61)	NA	NA	1,184 (26)
Lost-to-follow up	170 (9)	147 (11)	159 (12)	476 (10)
Died	55 (3)	58 (4.4)	27 (2)	140 (3)
Transferred	42 (2.1)	33 (2.5)	33 (2.4)	108 (2.3)
Treatment failure	8 (0.4)	NA	NA	8 (0.2)
Unknown	78(4)	31 (2.3)	32 (2.3)	140 (3)
Year of treatment				
2003	132 (7)	134 (10)	101 (7)	367 (8)
2004	153 (8)	101 (8)	132 (10)	386 (8)
2005	149 (8)	83 (6)	136 (10)	368 (8)
2006	145 (7)	93 (7)	107 (8)	345 (7)
2007	154 (8)	148 (11)	180 (13)	482(10)
2008	209 (11)	131 (10)	181 (13)	521 (11)
2009	184 (9)	129 (10)	119 (9)	432 (9)
2010	178 (9)	117 (9)	89 (7)	384 (8)
2011	373 (19)	124 (10)	165 (12)	662 (14)
2012	279 (14)	246 (19)	169 (12)	694 (15)

^a^For 13 cases TB classification was not mentioned

### Case notification rates

**Fig. 1:**
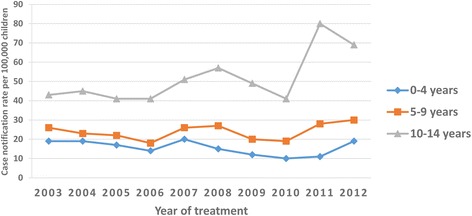
Case notification rates of TB of all forms among children in the Sidama Zone in southern Ethiopia, 2003–2012

The mean CNRs for new cases of TB of all forms were 30 per 100,000 children, 67 in urban and 28 in rural areas, and 32 in girls and 28 in boys per 100,000 children. No decline was observed in childhood TB over a ten-year study period (Fig. 1 and Table 3). The mean CNR of smear-positive PTB was about 13 per 100,000 children and increased from 11 in 2003 to 16 per 100,000 in 2012. The mean CNRs of smear-positive PTB were 14 for girls and 11 for boys per 100,000 children (Table 4).

**Table 3:** Characteristics of case notification rates and treatment outcomes of new cases of TB (4,545 cases) notified among children in the Sidama Zone, 2003–2012

Characteristics	CNR*/ 10^5^ people	Completed *N* (%)	Cured *N* (%)	Lost-to-follow up N (%)	Died *N* (%)	Transferred out *N* (%)	Treatment failure *N* (%)	Unknown *N* (%)
New cases of TB all forms	30	2532 (56)	1162 (26)	464 (10)	136 (3)	104 (2)	9 (0.2)	138 (3)
Sex								
Girls	32.0	1299 (51)	666 (28)	225 (10)	74 (3)	50 (2)	4 (0.2)	61 (3)
Boys	28.0	1233 (57)	496 (30)	225 (11)	62 (3)	54 (3)	5 (0.2)	75 (3)
Age category								
<5	15.3	459 (64)	47 (6.5)	128 (18)	44 (6)	21 (3)	1(0.1)	19 (3)
5–9	24.2	835 (60)	268 (19)	155(11)	41 (3)	37 (3)	4 (0.3)	53 (4)
10–14	53.0	1238 (51)	847 (35)	181 (8)	51 (2)	46 (2)	3 (0.2)	66 (3)
Residence								
Urban	67.0	368 (66)	88 (16)	54 (10)	16 (3)	13 (2)	1 (0.2)	14 (3)
Rural	28.0	2164 (54)	1074 (27)	410 (10)	120 (3)	91 (2)	8 (0.2)	124 (3)
TB classification								
Smear positive PTB	12.4	406 (21)	1161 (61)	158 (8)	53 (3)	39 (2)	8 (0.4)	77 (4)
Smear negative PTB	8.4	1013 (79)	NA	147 (12)	56 (4)	33 (3)	NA	30 (2.3)
EPTB	9.0	1112 (82)	NA	157 (12)	27 (2)	32 (2)	NA	31 (2)
Year of treatment								
2003	29.3	175 (48)	86 (24)	73 (20)	27 (7)	2 (1)	1 (0.3)	0
2004	28.5	199 (54)	72 (19)	54 (15)	16 (4)	15 (4)	0	15 (4)
2005	26.4	217 (60)	67 (19)	28 (8)	8 (2)	14 (4)	1 (0.3)	25 (7)
2006	23.7	197 (58)	56 (17)	45 (13)	11 (3)	7 (2)	0	23 (7)
2007	31.9	314 (66)	76 (16)	56 (12)	17 (4)	7 (2)	0	9 (2)
2008	32.7	309 (61)	111 (22)	60 (12)	9 (2)	7 (1)	1 (0.2)	8 (2)
2009	26.4	234 (56)	82 (20)	61 (15)	10 (2)	15 (4)	3 (1)	15 (4)
2010	23.0	198 (53)	76 (20)	60 (16)	10 (3)	16 (4)	0	16 (4)
2011	38.7	305 (47)	289 (44)	21 (3)	16 (3)	11(2)	1 (0.2)	9 (1)
2012	38.5	384 (57)	247 (36)	6 (1)	12 (2)	10 (2)	2 (0.3)	17 (3)
Retreatment cases	NA	27 (46)	18 (31)	6 (10)	3 (5)	3 (5)	0	2 (3)

*NA* not applicable

CNRs = the mean case notification rates over a ten-year period except for the year of treatment category

**Table 4:** Characteristics of case notification rates and treatment outcomes of new smear-positive PTB cases in children in the Sidama Zone, 2003–2012

Characteristics	CNR* per 10^5^	Completed (%)	Cured *N* (%)	Lost-to-follow up *N* (%)	Died *N* (%)	Transferred out *N* (%)	Treatment failure *N* (%)	Unknown *N* (%)
New PTB+ cases	12.6	406 (21)	1161 (61)	158(8)	53(3)	39(2)	8(0.4)	77 (2)
Sex								
Girls	14	229 (22)	665 (62)	85 (8)	29 (3)	17 (2)	3 (0.3)	38 (3.6)
Boys	11	177 (21)	496 (59)	73 (9)	24 (3)	22 (3)	5 (0.6)	38 (3.8)
Age category								
<5	3	38(30)	47(37)	24 (19)	10 (8)	6 (5)	0	2 (1.6)
5–9	8	107 (22)	267 (56)	48 (10)	14 (3)	9 (2)	4 (0.8)	30 (6.3)
10–14	28	261 (20)	847 (65)	86 (7)	29 (2)	24 (2)	4 (0.3)	45 (3.5)
Residence								
Urban	21	54 (31)	88 (51)	16 (9)	3 (2)	4 (2)	1 (0.6)	7 (4)
Rural	12	352 (20)	1073 (62)	142 (8)	50 (3)	35 (2)	7 (0.4)	70 (4)
Year of treatment								
2003	10.6	18 (14)	86 (65)	17 (13)	10 (8)	0	1 (0.8)	0
2004	11.4	41 (28)	72 (47)	19 (13)	4(3)	3 (2)	0	9 (6)
2005	10.5	44 (31)	67 (47)	7 (5)	5 (4)	4 (3)	1 (0.7)	16 (11)
2006	9.8	42 (30)	56 (40)	14 (10)	6 (4)	4 (3)	0	18 (13)
2007	10.2	49 (32)	76 (50)	20 (13)	3(2)	2 (1)	0	3 (2)
2008	12.7	59 (30)	111 (56)	19 (10)	1 (0.5)	4 (2)	1 (0.5)	2 (1)
2009	11.1	47 (27)	82 (47)	24 (14)	4 (2)	8 (5)	2(1)	9 (5)
2010	10.6	52 (30)	75 (43)	29 (17)	4 (2)	5 (3)	0	9 (5)
2011	21.7	44 (12)	289 (79)	7 (2)	6 (2)	6 (2)	1 (0.3)	6 (1.6)
2012	15.5	10 (3.7)	247 (91)	2 (1)	4 (2)	3 (1)	2 (0.7)	5 (2)

CNR* = mean case notification rate per 100,000 children except for the year of treatment category

### Treatment outcome

The proportions of treatment success were 82 % for new and 77 % for retreatment cases, and increased to 93 % in 2012 for new cases (*X*^2^ trend, *P* < 0.001) (Table 3). The proportion of cases lost-to-follow up declined from 20 % in 2003 to 1 % in 2012 (*X*^2^ trend, *P* < 0.001). Children less than five years old had a lower treatment success [AOR 0.64 (95 % CI, 0.52–0.80)] while the treatment success was higher in the 10-14 year-age group compared to 5–9 year olds [AOR 1.60 (95 % CI, 1.30–1.86)]. A considerable improvement was observed in treatment success in 2011–2012 (Table 5).

**Table 5:** Various factors associated with treatment success, loss-to-follow up and mortality among new cases of TB in children under 15 years in the Sidama Zone, 2003–2012

Characteristics of cases	Treatment success			Lost-to-follow up			Mortality		
	%	Adjusted OR (95 % CI)	*P*-value	%	Adjusted OR (95 % CI)	*P*-value	%	Adjusted OR (95 % CI)	*P*-value
Gender									
Girls	82.6	1.00		9.5	1.00		3.1	1.00	
Boys	79.9	0.84 (0.72–0.98)	0.028	11.0	1.26 (0.95–1.42)	0.136	2.9	0.90 (.64–1.28)	0.577
Age									
0–4 years	70.4	0.64 (0.52–0.80)	<0.001	17.8	1.68 (1.29–2.20)	<0.001	6.1	2.00 (1.27–3.12)	0.003
5–9 years	79.2	1.00		11.1	1.00		2.9	1.00	
10–14 years	85.7	1.60 (1.30–1.86)	<0.001	7.4	0.694 (0.55–0.86)	0.002	2.1	0.72 (0.47–1.10)	<0.129
<2 years	65.5	0.49 (0.35–0.69)	<0.001	18.1	1.58 (1.04–2.41)	0.032	10.5	3.34 (1.92–5.82)	<0.001
≥2 years	81.9	1.00		9.9			2.7	1.00	
Residence									
Urban	82.3	1.00		9.7	1.00		2.9	1.00	
Rural	81.1	0.83 (0.65–1.06)	0.131	10.3	1.20 (0.88–1.62)	0.265	3.0	1.08 (.65–1.92)	0.680
TB classification									
Smear positive PTB	82.4	1.00		8.3	1.00		2.8	1.00	
Smear Negative PTB	79.2	1.07 (0.88–1.31)	0.476	11.5	1.06 (0.82–1.40)	0.647	4.4	1.15 (0.76–1.75)	0.499
Extra pulmonary TB	81.8	1.18 (0.97–1.42)	0.098	11.6	1.18 (0.93–1.51)	0.181	2.0	0.58 (0.36–0.95)	0.029
Year of treatment									
2003	71.7	1.00		20.1	1.00		7.4	1.00	
2004	73.0	1.05 (0.76–1.46)	0.756	14.6	0.68 (0.46–1.01)	0.057	4.3	0.60 (0.32–1.14)	0.121
2005	78.9	1.44 (1.02–2.04)	0.038	7.80	0.34 (0.21–0.54)	<0.001	2.2	0.31 (0.14–0.69)	0.004
2006	74.6	1.14 (0.81–1.71)	0.443	13.3	0.60 (0.40–0.91)	0.016	3.2	0.46 (0.22–0.94)	0.033
2007	81.4	1.68 (1.12–2.34)	0.002	11.7	0.53 (0.36–0.76)	0.001	3.5	0.50 (0.27–0.93)	0.030
2008	83.2	1.90 (1.36–2.64)	<0.001	11.9	0.55 (0.38–0.81)	0.002	1.8	0.26 (0.12–0.57)	0.001
2009	75.2	1.11 (0.81–1.54)	0.517	14.5	0.73 (0.50–1.07)	0.105	2.4	0.34 (0.16–0.71)	0.004
2010	72.9	0.98 (0.71–1.37)	0.894	16.0	0.84 (0.57–1.22)	0.354	2.7	0.37 (0.18–0.78)	0.009
2011	91.1	3.64 (2.55–5.21)	<0.001	3.2	0.15 (0.09–0.25)	<0.001	2.5	0.38 (0.20–0.72)	0.003
2012	93.1	5.16 (3.54–7.52)	<0.001	0.9	0.037(0.02–0.09)	<0.001	1.8	0.24 (0.12–0.50)	<0.001

The proportion of childhood TB cases who died was 3 % (140 cases), constituting 12 % of the 1,202 deaths from TB in all age groups notified during the study period. The proportion of cases who died while on treatment declined from 7 % in 2003 to 2 % in 2012 (*X*^2^ trend, *P* < 0.001), and was higher among under five-year age group [AOR 2.00 (95 % CI, 1.27–3.12)], and less in EPTB cases [AOR 0.58 (95 % CI, 0.36–0.95)]. The proportion of children who died in the less than 2-year-age group was three times higher than in the 2 year and above age groups [AOR 3.34 (95 % CI, 1.92–5.82)].

The proportions of cases lost-to-follow up and who died while on treatment were less in the 10–14 year olds than the younger age groups. Notable improvements in treatment success, loss-to-follow up, and mortality were observed by years of treatment, and the highest improvement in treatment outcome was observed in 2011–2012 (Table 5).

## Discussion

We found low CNRs of childhood TB in Sidama. Our data show that the CNR in children was lower than other reports from developing countries [15, 16].

We analysed the CNRs based on age specific risk for developing TB, and challenges associated with its diagnosis [17, 18]. The low CNR among young children (less than 5 years old) in our finding could be explained by underreporting, difficulties in diagnosis of childhood TB due to distinct clinical features of the disease in young children [18], poor access to diagnostic facilities such as chest radiography and culture, and poor access to skilled health personnel to diagnose the disease in children. Instituting better diagnostic services such as interferon-gamma release assays, culture, and nucleic acid amplification tests could help improve diagnosis and treatment of childhood TB [19].

The higher CNRs among older children (10–14 years) in our data could be because the clinical features of the disease are often similar to adults, and there is a better yield of sputum-smear examination among older children [17, 20]. The higher CNRs in urban areas than in rural areas could be explained by better access to TB diagnostic facilities, better education and awareness of the parents and early health seeking, and this finding was in agreement with a study from Tanzania [21]. Likewise, in an earlier study, we found a higher CNR of TB among adults in urban areas [22].

The proportion of childhood TB cases in our data (13 %) was lower than the national estimate and report [2, 6]. Our finding was in agreement with a previous report from the study area (13 %) [23], and higher than the global report (6 %) [2]. Other studies also report childhood TB constitutes 10-20 % of the disease burden in endemic areas [17].

From Ethiopia, one study from an urban setting reported a lower proportion (6.6 %) [24], and another study from a rural hospital [25] reported a higher proportion (46 %) of childhood TB than our finding. The reported higher proportion could be because the study was conducted in hospital setting which could have better diagnostic setup for children and could be due to referral cases who come from high burden areas due to poor access to TB diagnosis and treatment facilities. The lower proportion of childhood TB for the former study [24] could be due to underreporting or due to the lower burden of the disease. Nevertheless, both studies did not report the CNRs of childhood TB per 100,000 children unlike our report. Thus, the differences in the proportion of childhood TB from both studies and our finding could be partly explained by differences in study settings because we conducted the study in both urban-rural settings, in all DOTS providing health facilities, and we linked TB cases to their true home address to compute the case notifications to avoid under- or over reporting [22].

In our study, we noted that more girls than boys had smear-positive TB in the 10–14-year age group. This could reflect, at least in part, that girls enter puberty earlier and therefore might develop adult type of PTB earlier than boys [19, 26]. In our data, the proportion of TB cases in children less than 5-year old was lower than in children 5–9-year old. In high burden setting with a broad-based population pyramid, the number of cases in children less than 5 and 10–14-year age groups are often higher than in the 5–9-year age group [4, 24]. The lower CNR in the younger age group in our study suggests TB in the less than 5-year age group could be underdiagnosed.

The proportion of smear-positive PTB among all pulmonary TB cases was higher than other reports from Ethiopia [24] and elsewhere [21, 27]. Evidence shows that children usually have approximately 10–15 % smear-positive TB [4]. The difference in our finding is most likely because of underdiagnosing of smear-negative cases, particularly in younger children. This suggests concerted efforts to improve the diagnoses of TB in the younger age group.

In our data, the proportions of children successfully treated were higher among older age groups than among younger children, as has been shown from other studies from Ethiopia [23, 24] and elsewhere [28]. Nevertheless, there is a growing concern on TB treatments in children, which are not “child-friendly” and TB drugs for children may not be in the correct dose, and the reports suggest “simple and safe treatments designed for children” [29]. The proportion of cases who died during treatment among children constituted about 12 % of the total deaths of TB in all age groups during the study period. However, the overall case fatality during treatment was 3 %, which is lower than a previous report from the study area [23].

We found that very young children (less than 2 years age) had poor treatment outcomes compared to children two and above years old, and this finding is consistent with other studies from Ethiopia, which report a higher mortality among young children [23, 24]. The possible reasons for poor treatment outcomes in younger children could be disseminated disease, poor or immature immunity, and late diagnosis of the disease. Many factors could also contribute to a higher risk of death in young children such as severe form of TB [1, 6], and co-morbidities such as malnutrition, HIV infection and pneumonia [4, 18, 24, 30]. Interventions such as contact tracing of adults with TB, which could help earlier diagnosis of the disease in the younger children is suggested to improve the treatment outcomes Additional file 1.

A study from Ethiopia conducted in urban setting reported an increased risk of mortality during treatment in the younger children and in children with TB/HIV co-infection [24]. We could not include the HIV status and the nutritional status of children in the risk analysis of treatment outcomes because the HIV and nutritional status of children were not recorded in the unit TB registers. Childhood malnutrition is a common health problem in the study area [10], and many children could be undernourished and this increases the risk of death among younger children.

Lastly, we found a noticeable reduction in mortality and loss-to-follow up and an increase in treatment success in 2011–2012. This could be due to community based interventions [11] and the accelerated expansion of TB diagnostic and treatment facilities in the study area [22, 31, 32], which have improved access to TB control services and early diagnosis of the disease. The regimen change of the continuation phase to rifampicin and isoniazid in 2011–2012, which have shortened the period of treatment, could also contribute to the observed decline in the proportion of lost-to-follow up cases among children.

The limitations of our study are that we could not confirm the diagnosis of the cases which may affect the case notification rates. We could not ascertain the HIV status of the children in our data to consider for the risk analysis of treatment outcomes; however, the prevalence of HIV in rural areas of southern region of Ethiopia is as low as 1 % [10], and may not significantly affect our results. We could not include the nutritional status of children because of inadequate data from the unit TB registers which could affect the treatment outcome. Misclassification of the diagnosis and treatment outcome is likely; however, most of the smear negative and extra pulmonary cases are diagnosed at hospitals which have better diagnostic facilities and health personnel than the peripheral health centres.

## Conclusion

We found that low CNRs of childhood TB and a higher mortality among younger children. Concerted effort should be made to improve diagnostic and treatment facilities for childhood TB as well as targeting intervention for high risk groups. Including HIV and nutritional status of the children in the risk analysis improves our understanding of predictors of mortality during treatment.

## Additional file

Additional file 1: Table S1. Age and sex characteristics of childhood tuberculosis cases in the Sidama zone in southern Ethiopia, 2003–2012. (DOCX 11 kb)
