# Supplementary material for: Low case notification rates of childhood tuberculosis in southern Ethiopia
Source: BMC Pediatr. 2015 Oct 1;15:142. doi: 10.1186/s12887-015-0461-1 (PMC4589978; doi:10.1186/s12887-015-0461-1)
Supplement: Additional file 1: Table S1. — Age and sex characteristics of childhood tuberculosis cases in the Sidama zone in southern Ethiopia, 2003–2012. (DOCX 11 kb) [file 12887_2015_461_MOESM1_ESM.docx]

**Additional file 1: Table S1 Age and sex characteristics of childhood tuberculosis cases in the Sidama zone in southern Ethiopia, 2003-2012**

| Characteristics | Smear positive PTB | Smear negative PTB | Extra pulmonary TB |
| --- | --- | --- | --- |
| **Boys** |  |  |  |
| 0-4 | 62 (48.8) | 177 (50.1) | 126 (52.9) |
| 5-9 | 213 (44.6) | 223 (50.5) | 236 (50.3) |
| 10-14 | 560 (43.2) | 242 (50.1) | 324 (49.7) |
| **Girls** |  |  |  |
| 0-4 | 65 (51.2) | 176 (49.9) | 112 (47.1) |
| 5-9 | 265 (55.4) | 219 (49.5) | 233 (49.7) |
| 10-14 | 736 (56.8) | 241 (49.9) | 328 (50.3) |

PTB= Pulmonary tuberculosis
